# Longitudinal assessment of chemotherapy-induced peripheral neuropathy in women undergoing taxane-based treatment for breast cancer: a prospective observational study

**DOI:** 10.1186/s12885-026-15750-8

**Published:** 2026-02-19

**Authors:** Rosiered Brownson-Smith, Jack Carr, Samuel T. Orange, Nicola Cresti, John M. Saxton, John Temesi

**Affiliations:** 1 Department of Nutritional Sciences, Faculty of Life Sciences & Medicine, King’s College London, London, England; 2 Department of Sport, Exercise and Rehabilitation, Faculty of Health and Life Sciences, Northumbria University, Newcastle-upon-Tyne, UK; 3 Research Centre for Physical Activity, Sport and Exercise Sciences, Coventry University, Coventry, UK; 4 School of Biomedical, Nutritional and Sport Sciences, Faculty of Medical Sciences, Newcastle University, Newcastle-upon-Tyne, UK; 5 Newcastle University Centre for Cancer, Newcastle University, Newcastle-upon-Tyne, UK; 6 Northern Centre for Cancer Care, The Newcastle upon Tyne Hospitals NHS Foundation Trust, Newcastle-upon-Tyne, UK; 7 School of Sport, Exercise & Rehabilitation Sciences, University of Hull, Kingston upon Hull, UK

**Keywords:** Breast cancer, Chemotherapy-induced peripheral neuropathy, Taxanes, Quality of life

## Abstract

**Background:**

Chemotherapy-induced peripheral neuropathy (CIPN) is a common, debilitating side effect of taxane-based chemotherapy. However, its trajectory during and after treatment remains poorly understood. This study aimed to estimate the natural history of CIPN symptoms using patient-reported and objective measures.

**Methods:**

Women with stage I-III breast cancer scheduled to receive taxane-based chemotherapy underwent CIPN assessments at five timepoints (pre-chemotherapy, post-anthracycline, mid-taxane, post-taxane, and 6-month follow-up) using physical function tests, patient-reported outcomes, quantitative sensory testing, H-reflex assessments, and static balance testing. Data were analysed using linear mixed models.

**Results:**

Twelve participants enrolled (48% of eligible patients, 100% retention). Self-reported autonomic (all timepoints), motor (post-anthracycline and post-taxane) and sensory (post-taxane and 6-month follow-up) symptoms were greater than pre-chemotherapy (all p < 0.05). Health-related quality of life was impaired at all timepoints compared to pre-chemotherapy. Maximal H-reflex amplitude normalised to maximal M-wave amplitude (H_max_/M_max_) decreased post-taxane (-0.23, 95% CI -0.36 to -0.10, p = 0.001) and at 6-month follow-up (-0.27, 95% CI -0.39 to -0.16, p < 0.001). Anterior-posterior (mid-taxane: 8 mm, 95% CI 2 to 13 mm, p = 0.007; post-taxane: 9 mm, 95% CI 4 to 15 mm, p = 0.001) and total (mid-taxane, p <0.001; post-taxane, p <0.001) sway increased from pre-chemotherapy, but mediolateral sway did not change (all timepoints p ≥ 0.05).

**Conclusions:**

Comprehensive CIPN assessment during and after chemotherapy is feasible and reveals impairments in nerve function and balance.

**Trial Registration:**

ClinicalTrials.gov Identifier: NCT05441722

**Supplementary Information:**

The online version contains supplementary material available at 10.1186/s12885-026-15750-8.

## Introduction

Paclitaxel and docetaxel are chemotherapeutic agents belonging to the taxane family [1]. Taxane-based regimens are amongst the most effective and commonly prescribed systemic therapies for early-stage breast cancer [2, 3]. The National Institute for Health and Clinical Excellence (NICE) recommends that taxanes are combined with anthracyclines for the treatment of lymph node-positive breast cancer in the adjuvant setting [4]. However, they can affect the structure and function of peripheral neurons, which can manifest as the neuropathic disorder chemotherapy-induced peripheral neuropathy (CIPN) [5–7].

It has been reported that all breast cancer patients experience at least one neuropathic symptom during docetaxel chemotherapy [8]. Also, 20–25% of breast cancer patients treated with paclitaxel or docetaxel report severe symptoms of CIPN by the end of treatment [5, 9]. CIPN impairs quality of life and can lead to dose reductions or discontinuation of chemotherapy [10, 11]. Symptoms can often persist for several months to years following treatment cessation [6, 7, 12, 13].

CIPN manifests as paraesthesia, dysesthesia, numbness, and tingling in a classic stocking and glove distribution [14]. CIPN can affect both the upper and lower limbs and with impairments in lower-limb sensory and motor function profoundly impacting balance and mobility and increasing the risk of falls, which can critically influence quality of life [15]. These symptoms can be quickly assessed with self-administered questionnaires, including the European Organisation for Research and Treatment of Cancer (EORTC) Quality of Life Questionnaire-CIPN 20-item scale (QLQ-CIPN20) [16–19]. However, although patient-reported symptoms of CIPN are reasonably well understood, many studies have been limited to a one-time assessment of outcomes [7, 8, 20], limiting our understanding of how CIPN symptoms change across taxane-based chemotherapy regimens.

The neurophysiological consequences of CIPN throughout the course of treatment are also poorly understood. Taxanes mainly damage large myelinated afferent fibres, and preclinical models have demonstrated that taxane chemotherapy induces dorsal root ganglion neuron degeneration in both peripheral and central sensory axons [21]. It has been proposed that this damage can interfere with proprioceptive cues, reducing the reliability of afferent feedback via sensory axons [22].

Electrophysiological techniques can be used to objectively assess relevant sensorimotor integration via the H-reflex and M-wave responses. Sensorimotor integration refers to the process by which sensory information, such as proprioceptive feedback, is received, processed, and used by the central nervous system to generate appropriate motor responses [23]. The H-reflex has previously been used to study peripheral neurological disorders via the H-reflex latency and ratio of maximal H-reflex (H_max_) to maximal M-wave (M_max_). For example, previous research has reported a slower lateral gastrocnemius H-reflex latency, relative to M-wave latency, in individuals with peripheral neuropathy versus healthy controls [24] and a cross-sectional study looking at CIPN in women with breast cancer found a smaller soleus H_max_/M_max_ in the neuropathy group versus healthy controls [22]. This research was able to identify differences between groups with and without CIPN. However, no studies have yet explored how H_max_/M_max_ and H-reflex latency evolve during neurotoxic chemotherapy. Investigating these changes may help determine whether the lower-limb H-reflex could serve as a useful tool for tracking the development and manifestation of CIPN.

Because CIPN has been linked to impairments in balance and an increased risk of falls [25], measurement of postural sway can also provide valuable insights into the extent of these deficits. An increase in sway area indicates balance control deficits and the risk of falling [26] but changes in sway path have not been regularly assessed throughout chemotherapy regimens. Accordingly, a reliable measure of postural stability is required to evaluate the relationship between potential changes in Ia afferent transmission at the spinal level and balance deficits. Furthermore, given the variability of assessment methods and the limited understanding of CIPN’s time course, it is important to evaluate measures at multiple time points to assess their feasibility and sensitivity for ongoing clinical monitoring.

This study primarily aimed to estimate the natural history of CIPN symptom burden, using both patient-reported and objective neurophysiological measures, across a taxane-based chemotherapy regimen. A secondary aim was to explore how these symptoms evolve and how best to monitor them.

## Methods

This was a longitudinal observational study, prospectively registered on ClinicalTrials.gov (NCT05441722). The development of this trial followed the Medical Research Council’s (MRC) published guidelines [27]. Reporting complied with the Strengthening the Reporting of Observational Studies in Epidemiology (STROBE) guidelines [28]. The completed STROBE checklist is available as Online Resource 1.

### Ethical approval

National Health Service (NHS) ethical approval from North East - Newcastle & North Tyneside 1 Research Ethics Committee was obtained on 6th March 2020 (ref: 19/NE/0341). Approval from the Health Research Authority (HCA) was granted on the 9th of March 2020. University ethical approval was granted by the Northumbria University Ethics Committee (reference: 40195).

### Research governance and good clinical practice

This study recruited participants receiving treatment for early-stage breast cancer from one NHS site. The researchers involved in the project received Good Clinical Practice training, and the study was conducted according to the Declaration of Helsinki (1964; revised 2001).

### Participant identification and recruitment

Patients with breast cancer were recruited from Gateshead Health NHS Foundation Trust (GHFT) by oncologist referral from May 2022 to March 2023. Potentially eligible participants were recruited during their initial oncology clinic appointment. They were initially approached by a Consultant Oncologist and given a participant information sheet. Inclusion criteria were women aged ≥ 18 years; histologically diagnosed stage IA-IIIC breast cancer; scheduled to receive adjuvant or neoadjuvant chemotherapy with sequential anthracycline-docetaxel/paclitaxel or weekly paclitaxel; willing and able to give written informed consent; and able to understand written and verbal instructions in English. Exclusion criteria included; pre-existing diabetes mellitus, pregnancy, cardiovascular, metabolic or renal disease; diagnosed with metastatic breast cancer; previous or existing symptoms of peripheral neuropathy; resting blood pressure (≥ 180/100 mmHg) or tachycardia (≥ 100 beats per min); any pre-existing musculoskeletal, neurological or psychiatric condition that may affect their ability to complete the testing battery; internal electrical regulator; previous exposure to taxanes at any time or exposure to other chemotherapy agents known to be associated with neuropathy within one year of starting in the study or currently enrolled in clinical trials involving exercise or pharmacotherapy that could influence CIPN symptoms. All participants gave written informed consent and were able to withdraw from the study at any time for any reason.

### Procedures

Outcomes were assessed at four or five time points, depending on the chemotherapy regimen that participants received. Each assessment took approximately 90 min. All participants completed outcome assessments at the following four time points: pre-chemotherapy (before starting chemotherapy), mid-taxane (after two infusions for those on block-sequential therapy, or after six infusions for participants receiving weekly paclitaxel), post-taxane (one to two weeks after completing all cycles of taxane-based chemotherapy, and 6-month follow-up (six months after completing taxane-based chemotherapy). Participants receiving block sequential anthracycline-docetaxel/paclitaxel underwent an additional assessment after completing anthracycline therapy (post-anthracycline). Pre-chemotherapy assessments established baseline function. The post-anthracycline timepoint allowed us to identify any neurotoxic effects of anthracyclines [29]. Mid- and post-taxane assessments captured the development and cumulative impact of neurotoxicity, which has been shown to accumulate over the course of treatment [30]. Follow-up assessments evaluated recovery or persistence of symptoms, reflecting the phenomenon of ‘coasting,’ where neuropathic symptoms continue after treatment completion [31]. A schematic of the study design is found in Online Resources 2. All data were collected at either the Queen Elizabeth Hospital, Gateshead or Northumbria University.

To minimise potential sources of bias, objective physiological measures were conducted using validated instruments and consistent protocols across all visits. Efforts to minimise attrition bias included flexible scheduling and regular participant contact.

### Sample size

Due to the exploratory nature of this pilot study, no formal sample size calculation was performed [32]. Guidelines for pilot-controlled trials recommend at least 12 participants for each study arm [31].

### Outcome measures

#### Feasibility outcomes

This trial focused on determining the feasibility of recruitment and data collection. Recruitment rate was determined from the number of eligible participants who were invited and those that gave informed consent to participate in the trial. Retention rate was calculated as the proportion of participants who completed all follow-up assessments compared to those who initially consented to participate. Data completeness was reported as the percentage of expected data points successfully collected across all outcome measures and time points.

#### Patient-reported outcomes

Self-reported symptoms of CIPN were measured with the EORTC QLQ-CIPN20 [33]. This questionnaire comprised 20 items where patients rated their experience with each symptom over the past week. Item 20 assessed erectile dysfunction; therefore, as this study included only women, this item was excluded from the CIPN20 score. The Functional Assessment of Chronic Illness Therapy-Fatigue (FACIT-Fatigue) questionnaire [34] was administered to assess fatigue. Disease-specific health-related quality of life (HR-QOL) was assessed using the Functional Assessment of Cancer Therapy-Breast (FACT-B) [35]. Pain was measured with the 20-item Pain Quality Assessment Scale (PQAS) [36]. Self-reported level of physical activity was evaluated using the Godin Leisure-Time Exercise Questionnaire (GLTEQ) [37], which measured the frequency of strenuous, moderate, and mild exercise performed for at least 15 min per session over a typical week multiplied by a corresponding MET (Metabolic Equivalent of Task) value to quantify the intensity of the activity.

#### Quantitative sensory testing

A range of quantitative sensory testing (QST) methods were used to measure distal sensory neuropathy in both feet. All QST techniques were conducted whilst participants had their eyes closed.

#### Vibration perception

Vibration perception was measured using a 128-Hz tuning fork. The “on-off” method was used. The tuning fork was struck before being applied to the bony prominence on the dorsum of the hallux in the feet, just proximal to the nail bed. Participants indicated whether they felt a vibration and when it stopped. One point was assigned for each vibration sensation correctly detected, and another point for correctly timing the vibration’s dampening [38].

#### Touch perception

Touch perception was assessed using a Semmes-Weinstein 10-g Monofilament. The monofilament was pressed against the skin so that it buckled into a C shape for 3 s. The participant was asked to indicate whether they felt the touch. Four sites were tested on each foot: the plantar surface of the hallux, the plantar surfaces of the first, third and fifth metatarsal heads [39, 40].

#### Temperature perception

Temperature perception was measured using a Tip-therm (Tip Therm GmbH, Brueggen, Germany). The pen-like device has a metal and polymer cylinder [41]. The metal side feels colder due to thermal conductivity. Both ends were applied to the dorsal foot between the hallux and second toe for 3 s [42]. Participants indicated which surface felt colder. Correct perceptions earned one point.

### Physical functioning

#### Short Physical Performance Battery (SPPB)

The SPPB is a composite measure of standing balance, gait speed and chair sit-to-stand tests [43]. The tests included: (1) The ability to stand for up to 10 s with feet positioned side-by-side, semi-tandem and tandem (with the dominant leg forward). (2) Time to complete a 4-m walk at usual pace. (3) Number of sit-to-stands achieved in 30 s. The chair sit-to-stand component of the SPPB was conducted using a 30-s-Chair-Stand-Test, in which participants were asked to complete as many full sit-to-stand cycles as possible within 30 s. This protocol has been found to be more feasible and a superior predictor of physical function than the 5-times-repeated-Chair-Stand-Test [44].

#### Tandem walk test

Participants walked for 10 steps, heel-to-toe, without spaces between the steps. Two trials were performed, one with eyes open and one with eyes closed. The maximum number of consecutive steps was recorded (maximum of 10). Errors included taking a sidestep, making a space between the feet, and opening the eyes during the eyes closed trial [45].

#### Maximal isometric grip strength

Participants squeezed an analogue hand grip dynamometer (TKK 183 5001 Grip-A, Takei Scientific Instruments Ltd., Tokyo, Japan) as hard as possible for 2–3 s. Three maximal trials were performed on each hand, with the highest score used for analysis.

### Sway

#### Accelerometer

A wireless sensor (Trigno^®^ Wireless Biofeedback System, Delsys Inc., Manchester, UK) was attached to the participants’ lower back at the level of the posterior superior iliac spine. While participants performed a 30-s static bipedal balance test barefoot with feet shoulder-width apart, accelerations were sampled at 148 Hz by tri-axial accelerometery and recorded in Labchart 8 (ADInstruments, Oxford, UK) via Trigno Control Utility plug-in (Delsys Inc.). Offline, data was analyzed using Labchart 8 software where a 4th-order Butterworth filter with cut-off frequency of 1.25 Hz was applied [44]. Normalized path length (NPL) was then calculated in the anterior-posterior (AP) and mediolateral (ML) directions as [46]:

$$\:NPL=\:\frac{1}{t}\sum\:_{j=1}^{N-1}|{p}_{j+1}-{p}_{j}|$$

where *t* is the time duration, *N* is the number of time samples, and *p*_*j*_ is the accelerometer data at time *j*.

#### Swaymeter

Participants wore a swaymeter, a belt with an inflexible rod and a vertically mounted pen, at the level of the posterior superior iliac spine. The pen traced a line on mm graph paper, recording the body’s displacement. Participants stood barefoot and still for 30s on both legs, with an initial recording for familiarisation. The sway path length was manually counted, and peak-to-peak sway displacements in the AP and ML directions were calculated based on the extremes of sway length in these planes [47, 48].

#### Anthropometry

Body mass was measured to the nearest 0.1 kg using a calibrated digital scale. Standing height was measured to the nearest 0.1 cm with a free-standing stadiometer. Waist and hip circumferences were measured to the nearest 0.1 cm using an anthropometric measure tape.

#### Sensorimotor integration

While participants were standing barefoot with both feet in line with their shoulders, single-pulse electrical stimulation (1-ms pulse duration) were delivered by constant-current electrical stimulator (DS7A, Digitimer, Welwyn Garden City, Hertfordshire, UK) to the right tibial nerve via stimulating bar electrode with 30-mm anode-cathode spacing (E.SB020/4 mm Bipolar Felt Pad Stimulating Electrode, Digitimer) for H-reflex optimization [49]. Electromyography of the soleus was recorded with a pair of self-adhesive surface (10-mm recording diameter) electrodes (Meditrace 100, Covidien, Mansfield, MA) in bipolar configuration with 30-mm interelectrode distance for optimal H_max_ recording [50] and the reference on the medial malleolus to record H reflexes and M waves. Beginning at 5 mA, stimuli were delivered at least 10-s apart with increasing increments of 1 mA, until 40 mA or a plateau in the M-wave amplitude was reached, whichever occurred later. Electromyographic signals were sampled at 2000 Hz using a PowerLab 8/35 data acquisition system and quad bio-amplifier (ADInstruments) with band pass (5–500 Hz) and notch (50 Hz) filters and analysed offline using Labchart 8 software (ADInstruments).

#### Statistical analysis

Descriptive statistics are presented for participant characteristics and feasibility outcomes (e.g., recruitment, retention). Linear mixed modelling (LMM) was used to estimate the change in outcome measures over time. Timepoint was a fixed effect with five levels: pre-chemotherapy, post-anthracycline, mid-taxane, post-taxane, and 6-month follow-up. Pre-chemotherapy was the control timepoint. Post- anthracycline data was missing when there were only four time points (weekly paclitaxel). A random intercept-only model was used to account for the hierarchical nature of the data (repeated measures nested within participants), allowing for individual variations in baseline measures while maintaining a common slope. Models were fit using maximum likelihood. Residual normality was assessed both visually (Q-Q plots) and statistically using the Shapiro-Wilk test. LMMs handle missing data under the assumption of missing at random (MAR), where the probability of missingness depends on observed data but not unobserved data. In two cases, post- anthracycline data were missing because the weekly paclitaxel-only chemotherapy regimen did not include an anthracycline phase. This resulted in structurally missing data. However, this occurred in a small subset of the sample, and LMM was still considered the most appropriate method due to its robustness and ability to include all available data. The model incorporated all available data points to maximise information use and avoid bias from complete-case analysis [51]. A sensitivity analysis was conducted by including treatment timing (neoadjuvant vs. adjuvant) as an additional fixed effect in the LMM to assess whether this factor influenced the trajectory of outcomes. Significance was set at *p* < 0.05. Estimates of fixed effects were calculated as estimated marginal means from LMM, reflecting adjusted mean changes from pre-chemotherapy at each timepoint with corresponding 95% confidence intervals. All analyses were conducted in R (R Foundation for Statistical Computing, Vienna, Austria, version 4.3.1).

### Results

**Fig. 1:**
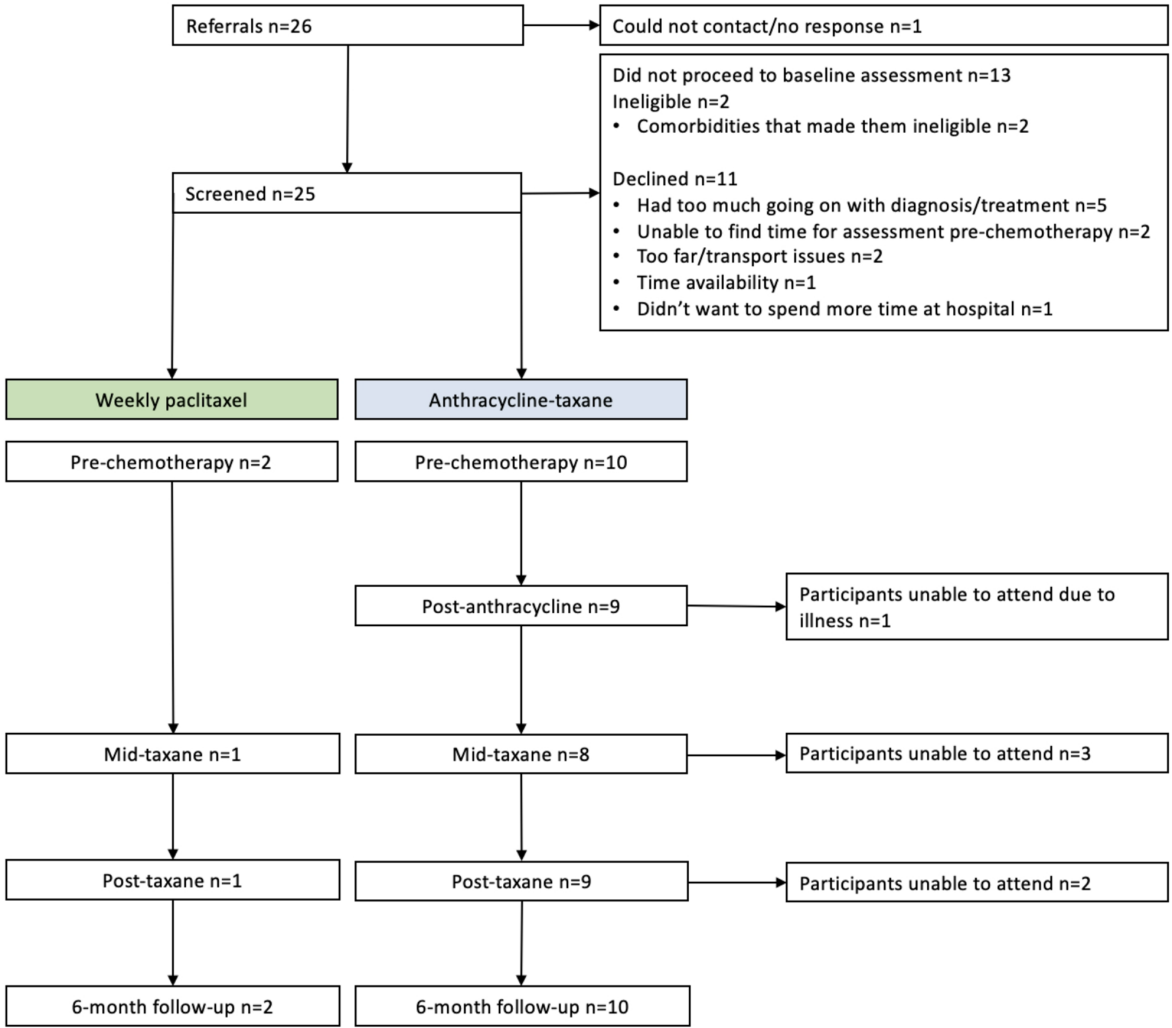
STROBE flow chart of participants going through the trial

Between May 2022 and March 2023 twenty-six patients were approached about participation in the study and 12 enrolled and completed pre-chemotherapy testing (Fig. 1). Participant characteristics at the pre-chemotherapy visit are shown in Table 1.

**Table 1:** Sociodemographic and clinical characteristics of participants at the pre-chemotherapy visit

Characteristics	*N* = 12
Age (years) (mean ± SD)	50 ± 8
Body mass (kg) (mean ± SD)	83.8 ± 16.8
Height (cm) (mean ± SD)	165.0 ± 4.0
Partner (yes)	11 (92%)
Education level	
None or primary school	0 (0%)
Lower general secondary education or vocational training	6 (50%)
Pre-university education, high vocational training, university	5 (42%)
Not disclosed	1 (8%)
Employed	10 (83%)
Retired	2 (17%)
Ethnicity	
White British	12 (100%)
Smoking status (yes)	1 (8%)
Number of comorbidities	
None	6 (50%)
One	5 (42%)
Two or more	1 (8%)
Treatment regime	
Weekly paclitaxel	2 (17%)
Block anthracycline -paclitaxel	2 (17%)
Block anthracycline -docetaxel	8 (67%)
Chemotherapy	
Adjuvant	9 (75%)
Neoadjuvant	3 (25%)

*Variables may deviate from 100% due to rounding off. Abbreviations: SD = standard deviation*

### Feasibility outcomes

Study recruitment rate was 48%. All participants who consented and completed pre-chemotherapy testing also attended the 6-month follow-up visit (100% retention rate). However, the completion rates for individual measures varied due to requirements such as wearing shorts, additional space needs, and hardware issues encountered at NHS site. The completion rates for all planned testing sessions (58) were as follows: anthropometry (84%), standing time (90%), 4-m walk (86%), sit-to-stand (90%), tandem walk (90%), grip strength (86%), patient-reported outcomes (90%), quantitative sensory testing (86%), sway (swaymeter = 86%, accelerometer = 84%), and sensorimotor integration (72%).

### Anthropometry

Mean for each measure (± standard deviation) at each timepoint are shown in Online Resource 3. There were no statistically significant changes in body mass throughout chemotherapy, or at follow-up. However, there was a decrease in waist circumference, relative to pre-chemotherapy, at follow-up (Online Resource 4). Additionally, hip circumference was found to have increased from pre-chemotherapy levels at the mid-taxane and post-taxane visits (Online Resource 4).

### Patient reported outcomes

Change in mean scores (± standard deviation) at each timepoint are shown in Table 2. An increase in patient-reported sensory CIPN, as measured by the EORTC QLQ-CIPN20, from pre-chemotherapy levels was found at the post-taxane and 6-month follow-up visits. Motor CIPN, measured by the EORTC QLQ-CIPN20, increased from pre-chemotherapy at the mid-taxane, post-taxane, and 6-month follow-up visits. Autonomic CIPN symptoms were significantly elevated at all timepoints compared to pre-chemotherapy. The FACT-B score was diminished in comparison to pre-chemotherapy levels at all timepoints except the 6-month follow-up. The FACIT Fatigue Score showed a decrease relative to pre-chemotherapy at all timepoints, indicating an increase in fatigue during chemotherapy. Statistically significant increases in total PQAS scores were observed at the post-taxane and follow-up visits. There were no statistically significant changes in activity levels across the chemotherapy cycle, as measured by the GLTEQ outcome. Means for each measure (± standard deviation) at each timepoint are shown in Online Resource 3.

**Table 2:** Longitudinal changes from baseline (pre-chemotherapy) in patient-reported, quantitative sensory testing, neurophysiological and sway measures

Outcome Measure	Timepoint	Change from pre-chemotherapy (mean, 95% CI)	*p*-value
EORTC QLQ-CIPN20 Sensory	Post-anthracycline	2 (-1–5)	0.216
	Mid-taxane	3 (-1–6)	0.123
	Post-taxane	6 (3–9)	< 0.001
	6-month follow-up	7 (4–10)	< 0.001
EORTC QLQ-CIPN20 Motor	Post-anthracycline	2 (0–3)	0.078
	Mid-taxane	2 (0–4)	0.033
	Post-taxane	3 (2–5)	< 0.001
	6-month follow-up	4 (2–5)	< 0.001
EORTC QLQ-CIPN20 Autonomic	Post-anthracycline	1 (0–2)	0.016
	Mid-taxane	1 (0–2)	0.001
	Post-taxane	1 (0–2)	0.002
	6-month follow-up	1 (0–2)	0.002
FACIT Fatigue Scale	Post-anthracycline	-16 (-23 – -9)	< 0.001
	Mid-taxane	-18 (-26 – -11)	< 0.001
	Post-taxane	-16 (-23 – -9)	< 0.001
	6-month follow-up	-6 (-13–0)	0.057
FACT-B	Post-anthracycline	-14 (-27 – -1)	0.029
	Mid-taxane	-18 (-31 – -6)	0.005
	Post-taxane	-17 (-29 – -5)	0.008
	6-month follow-up	-2 (-14–9)	0.688
PQAS Total	Post-anthracycline	1 (0–2)	0.062
	Mid-taxane	0 (-1–2)	0.439
	Post-taxane	1 (0–2)	0.034
	6-month follow-up	2 (0–3)	0.006
GLTEQ	Post-anthracycline	-8 (-22–6)	0.235
	Mid-taxane	-12 (-27–3)	0.104
	Post-taxane	-12 (-26–1)	0.074
	6-month follow-up	8 (-5–21)	0.245
Vibration (x/8)	Post-anthracycline	0 (-1–1)	0.950
	Mid-taxane	0 (-1–1)	0.489
	Post-taxane	-1 (-1–0)	0.085
	6-month follow-up	0 (-1–0)	0.287
Touch (x/8)	Post-anthracycline	0 (0–0)	0.896
	Mid-taxane	0 (-1–0)	0.831
	Post-taxane	0 (-0–0)	0.416
	6-month follow-up	0 (-1–0)	< 0.001
Temperature (x/4)	Post-anthracycline	0 (-0–1)	0.531
	Mid-taxane	0 (-1–0)	0.508
	Post-taxane	1 (0–1)	0.106
	6-month follow-up	0 (-1–1)	0.632
M_max_ (mV)	Post-anthracycline	-0.46 (-1.48–0.56)	0.366
	Mid-taxane	-0.68 (-1.66–0.30)	0.167
	Post-taxane	-1.40 (-2.39 – -0.42)	0.007
	6-month follow-up	3.85 (2.94–4.77)	< 0.001
H_max_ (mV)	Post-anthracycline	-0.63 (-1.84–0.57)	0.293
	Mid-taxane	-0.36 (-1.52–0.80)	0.533
	Post-taxane	-0.44 (-1.61–0.73)	0.449
	6-month follow-up	-4.73 (-5.82 – -3.64)	< 0.001
H_max_/M_max_	Post-anthracycline	-0.02 (-0.15–0.11)	0.768
	Mid-taxane	-0.11 (-0.23–0.01)	0.078
	Post-taxane	-0.23 (-0.36 – -0.10)	0.001
	6-month follow-up	-0.27 (-0.39 – -0.16)	< 0.001
H-reflex Latency (ms)	Post-anthracycline	0 (-2–2)	0.892
	Mid-taxane	2 (0–4)	0.083
	Post-taxane	2 (0–4)	0.022
	6-month follow-up	4 (2–5)	< 0.001
Swaymeter - ML Displacement (mm)	Post-anthracycline	3 (-2–9)	0.203
	Mid-taxane	1 (-4–6)	0.640
	Post-taxane	2 (-4–7)	0.563
	6-month follow-up	2 (-3–7)	0.479
Swaymeter - AP Displacement (mm)	Post-anthracycline	2 (-3–7)	0.474
	Mid-taxane	8 (2–13)	0.007
	Post-taxane	9 (4–15)	0.001
	6-month follow-up	1 (-4–7)	0.600
Swaymeter - Total Sway (mm)	Post-anthracycline	30 (-1–60)	0.059
	Mid-taxane	54 (24–85)	< 0.001
	Post-taxane	55 (24–85)	< 0.001
	6-month follow-up	26 (-4–57)	0.090
ML NPL (mG/s)	Post-anthracycline	− 0.38 (-1.27–0.50)	0.386
	Mid-taxane	-0.03 (-0.92–0.85)	0.943
	Post-taxane	-0.83 (-1.63 – -0.02)	0.044
	6-month follow-up	0.09 (-0.72–0.91)	0.818
AP NPL (mG/s)	Post-anthracycline	-1.78 (-5.01–1.46)	0.274
	Mid-taxane	-1.56 (-4.80–1.67)	0.336
	Post-taxane	-1.91 (-4.85–1.03)	0.197
	6-month follow-up	2.12 (-0.87–5.11)	0.160

Changes from the pre-chemotherapy timepoint. Estimates of fixed effects are reported as changes from baseline (pre-chemotherapy), with corresponding 95% confidence intervals (CIs). Positive estimates indicate an increase from baseline, and negative estimates indicate a decrease. Values in bold are statistically significant (*p* < 0.05). Abbreviations: EORTC QLQ-CIPN20 = European Organisation for Research and Treatment of Cancer Quality of Life Questionnaire - Chemotherapy-Induced Peripheral Neuropathy 20-item module, FACIT-Fatigue = Functional Assessment of Chronic Illness Therapy - Fatigue Subscale, Fact-B = Functional Assessment of Cancer Therapy - Breast, PQAS = Pain Quality Assessment Scale, GLTEQ = Godin Leisure-Time Exercise Questionnaire, M_max_ = maximum amplitude of the M-wave, H_max_ = maximum amplitude of the H-reflex, H-reflex latency = time interval from stimulation onset to the H-reflex, ML = mediolateral, AP = anterior-posterior

### Physical functioning 

There were no statistically significant changes in any physical functioning measure (Table 2). Additionally, all participants were able to complete all 10 s of the side-by-side and semi-tandem standing times, and only one participant was unable to complete all 10 s in tandem standing at the mid-taxane assessment. Mean for each measure (± standard deviation) at each timepoint are shown in Online Resource 3.

### Quantitative sensory testing

There were no statistically significant changes in the sensory vibration or temperature perception measures. The mean touch perception score remained consistent throughout the chemotherapy cycle, however, there was a statistically significant decrease from pre-chemotherapy at the 6-month follow-up visit (Table 2). Mean for each measure (± standard deviation) at each timepoint are shown in Online Resource 3.

### Sensorimotor integration

**Fig. 2:**
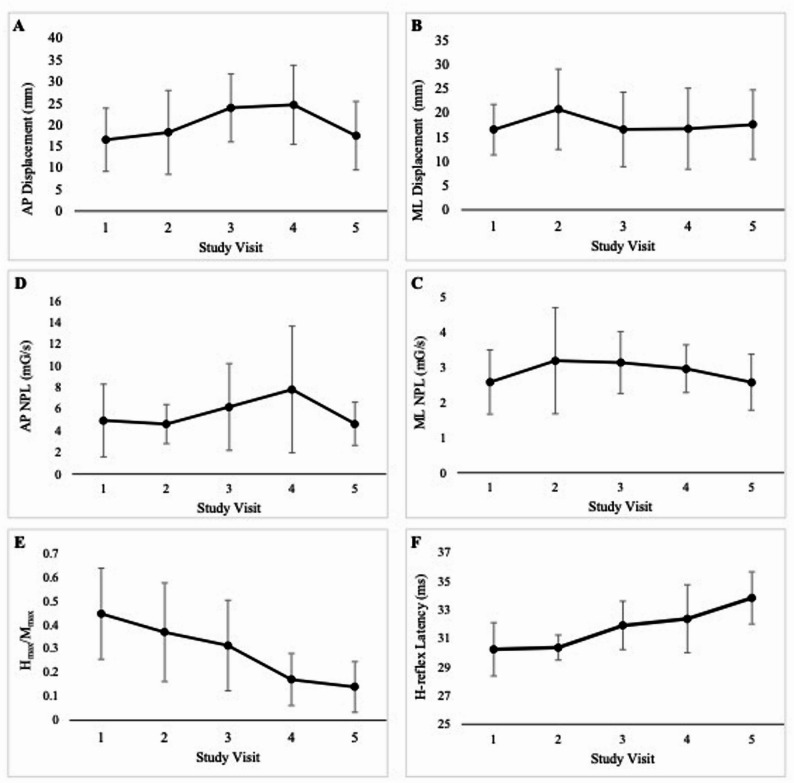
Sway and sensorimotor outcomes over time. **A** Anterior-posterior (AP) displacement measured via the swaymeter. **B **Mediolateral (ML) displacement measured via the swaymeter. **C **Normalised path length (NPL) in the AP direction, calculated using accelerometery data. **D **Normalised path length (NPL) in the ML direction, calculated using accelerometery data. **E **Maximum amplitude of the H-reflex over the maximum amplitude of the M-wave (Hmax/Mmax) **F**) Latency of the H-reflex. Numerical values along the y-axis are associated with the following timepoints: 1 = pre-chemotherapy, 2 = post-anthracycline, 3 = mid-taxane, 4 = post-taxane, 5 = 6-month follow-up. Data presented are mean ± standard deviation. Abbreviations: Mmax = maximum amplitude of the M-wave, Hmax = maximum amplitude of the H-reflex, H-reflex latency = time interval from stimulation onset to the H-reflex, ML = mediolateral, AP = anterior-posterior

Mean and standard deviation across timepoints are presented graphically in Fig. 2. Mmax decreased from pre-chemotherapy at the post-taxane timepoint but then increased from pre-chemotherapy levels at follow-up. Hmax was lower than baseline at 6-month follow-up. Hmax/Mmax was lower at post-taxane and the 6-month follow-up compared to pre-chemotherapy (Table 2). Similarly, H-reflex latency did not deviate from pre-chemotherapy levels at the post-anthracycline and mid-taxane visits, but a significant decrease from pre-chemotherapy levels was observed at the post-taxane and 6-month follow-up visits (Table 2). Means for each measure (± standard deviation) at each timepoint are shown in Online Resource 3.

### Sway

Mean and standard deviation across timepoints for sway are presented graphically in Fig. 2. AP displacement increased significantly from pre-chemotherapy at the mid-taxane and post-taxane visits. AP displacement then returned to pre-chemotherapy levels at the 6-month follow-up visit (Table 2). However, no deviations from pre-chemotherapy were observed in ML displacement. Finally, the total sway increased from pre-chemotherapy levels at the mid-taxane and post-taxane visits (Table 2). Again, there were no significant changes from pre-chemotherapy at follow-up. ML NPL remained unchanged from baseline at post-anthracycline and mid-taxane but decreased post-taxane. It then returned to baseline levels at follow-up. AP NPL did not change from baseline at any timepoint (Table 2).

### Sensetivity analysis 

For almost all measures, secondary sensitivity analysis, including treatment timing (neoadjuvant vs. adjuvant) as a fixed effect, did not significantly improve model fit. However, it did improve model fit for Hmax and FACIT-Fatigue, suggesting some impact of timing on these measures.

## Discussion

This study revealed several novel and important findings. Firstly, assessment of CIPN via various objective and subjective outcomes throughout and after taxane chemotherapy is feasible, although some objective physiological measurements could not be completed in all participants. The results demonstrated that chemotherapy significantly affected a range of outcomes. These included sensory, motor, and autonomic symptoms of CIPN, as well as pain, fatigue, HR-QOL, static balance, and peripheral nerve function. While some symptoms, such as balance and HR-QOL, returned to pre-chemotherapy levels post-treatment, others, such as fatigue and peripheral nerve function, showed persisting effects.

### Feasibility

The recruitment rate of this study suggests that, despite the additional time burden of the assessments, there was a willingness to take part in this research, indicating the potential success of future studies. Although data completeness varied across measures, it generally remained high. Peripheral nerve function testing had the lowest completeness (72%) due to test tolerance, symptom side effects, and hardware issues on-site. The primary reasons for missed mid- and post-chemotherapy appointments were chemotherapy side effects, including fatigue and infection. While surface electromyography is non-invasive, the intensity of electrical stimulation to elicit Mmax caused some discomfort, leading to early test termination in two cases.

### Patient-reported outcomes

Patient-reported sensory CIPN symptoms increased from pre-chemotherapy levels at post-taxane and 6-month follow-up, with changes exceeding the threshold for clinically meaningful change [52]. Taxane-induced sensory changes in the hand and foot are common post-paclitaxel and docetaxel chemotherapy [53-55]. Worsening of symptoms recorded by all EORTC subscales align with prior research into taxane chemotherapy [56]. Sensory neurons are more sensitive to taxane-induced damage, although the exact mechanism remains unclear [57]. This study found significant increases in autonomic and motor symptoms at earlier timepoints (post-anthracycline and mid-taxane); however, the change in motor symptoms did not reach the threshold for clinical meaningfulness [52], and no such threshold has yet been established for the autonomic subscale. The origins of these symptoms may belong within the direct consequences of a cancer diagnosis, and the receipt of chemotherapeutic agents prior to taxanes. Autonomic symptoms include dizziness and blurred vision, which may result from direct neurotoxicity of anthracyclines or anthracycline-induced vascular damage. Chemotherapy-induced changes in blood pressure may contribute to cardiotoxicity-related symptoms such as blurred vision and dizziness [58]. Motor-related symptoms may develop because of systemic cancer related fatigue, or physical deconditioning during chemotherapy [59, 60].

HR-QOL declined throughout chemotherapy, with impairments exceeding the threshold for clinical meaningfulness from post-anthracycline to post-taxane and persisting after treatment [61]. However, mean HR-QOL returned to pre-chemotherapy levels by the follow-up assessment. Previous research has shown negative effects of chemotherapy on breast cancer HR-QOL [62] with similar returns to baseline level reported [63]. For patients receiving neoadjuvant chemotherapy, pre-chemotherapy scores may have been influenced by the psychological burden of a recent cancer diagnosis. For adjuvant patients, the baseline assessment followed surgery, and scores may have reflected the ongoing physical recovery and emotional distress associated with post-operative healing [64, 65].

This study observed an increase in mean total PQAS scores at post-taxane and follow-up visits. CIPN is likely a contributor, but taxane-induced pain is complex and could have been caused by muscle and joint pain, acute neuropathic pain syndrome, or concomitant treatments [13, 66].

### Sensorimotor integration

Taxane chemotherapy-induced Hmax/Mmax and H-reflex latency changes suggest decreased motor neuron recruitment or reduced synaptic efficacy within the reflex arc, potentially leading to suboptimal muscle activation for balance control. However, this interpretation may be oversimplistic, as multiple factors influence characteristics of the H-reflex. Reductions in Hmax amplitude can also result from increased M-wave activity causing antidromic collision in motor axons, independent of changes in synaptic efficacy [67].

Taxanes damage large myelinated afferent fibres, such as Ia fibres, which play a crucial role in maintaining balance by providing continuous feedback on muscle length and stretch [22]. Changes in Ia afferent transmission at the spinal level may be responsible for gait and balance disorders, by delaying and/or reducing the magnitude of afferent feedback [68]. Slow conduction velocity, a feature of taxane-induced polyneuropathy, indicates a longer time between stimulation and response, resulting in a prolonged latency [69]. These effects likely arise from taxanes' microtubule-binding action that disrupts neuronal structure and function [70].

### Sway

When measured with the swaymeter, total and AP displacement increased significantly during and after taxane chemotherapy, while ML displacement remained unchanged. In contrast, NPL remained stable in the AP direction and decreased at post-taxane in the ML direction. This discrepancy may be due to methodological differences: swaymeter data represent the maximal displacement of a pen tracing sway on a surface, whereas accelerometer-derived NPL quantifies the total path length travelled in each direction. This difference highlights the need for further investigation to understand true changes in postural control. Furthermore, the lack of a gold-standard comparator, such as a force plate [71, 72], limited our ability to resolve this discrepancy. However, both methods detected no difference from pre-chemotherapy at the 6-month follow-up visit, indicating return to pre-chemotherapy levels. The results of the current study differ from the body of published literature where changes in balance and sway are primarily reported in the mediolateral direction [56, 73-78]. Of those studies that assessed direction-dependent sway changes in both directions, three found deficits in mediolateral sway [22, 73, 78], and two also reported an increase in AP sway [22, 73]. However, there is considerable heterogeneity in the methodologies used to assess and quantify sway across studies. Various techniques have been employed, including force plates, custom-made motion capture systems, and wearable devices. This may have contributed to differences in reported outcomes and complicate direct comparisons between studies.

Objective measures of postural sway have consistently reported compromised postural stability among cancer survivors [56, 78, 79]. Additionally, across a range of clinical populations, peripheral neuropathy has been linked to increased postural sway and increased falls [80, 81]. The increase in sway observed in individuals undergoing taxane-based chemotherapy may be attributed to CIPN, as with the decrease in Hmax/Mmax and the increase in H-reflex latency. Lower normalised H-reflex amplitude reflects diminished spinal reflex response. The increased latency may also indicate a delayed neural conduction. The greater sway increase in the AP direction likely reflects control by lower leg muscles, especially the soleus, which, along with the gastrocnemius, generates plantar flexion to counter forward sway [82]. In contrast, ML balance relies more on hip abductors and adductors [83].

This study reveals a complex relationship between sway and H-reflex changes: sway impairment appeared earlier (mid-treatment), while H-reflex deficits emerged only post-treatment. Sway had recovered by the 6-month follow-up, yet H-reflex deficits persisted. Sensory symptoms aligned with H-reflex changes, suggesting sensory nerve involvement. These findings imply sway changes result from multiple factors, including muscle strength and neuromuscular mechanisms. Muscle strength decline often precedes neurophysiological deficits [84], contributing to early impairments. While physical activity levels remained unchanged in the present study, the GLTEQ did not track sedentary behaviour, which is higher in breast cancer survivors [85, 86] and linked to falls [87]. Increased sedentary time post-diagnosis may contribute to early sway changes, with recovery driven by muscle function improvements despite nerve deficits. Muscle strength correlates with static balance control [88], supporting post-treatment recovery through lifestyle adjustments.

### QST

Impaired detection of touch was observed at the follow-up assessment. Myelinated fibres normally convey light touch sensation. Impairment of this modality suggests selective damage to these fibres [54]. However, although changes in vibration and temperature perception were not observed in the present study, QSTs were not assessed continuously and may have lacked sensitivity. Objective sensory outcomes in future studies in this population should aim to increase test sensitivity, potentially via a continuous methodology where the stimulus is either increased or reduced.

Additionally, the included methods for assessing sensory changes within the periphery that were included in this study were limited to the feet. As CIPN presents in a glove and stocking distribution, future studies should assess potential sensation changes within the hands that were not captured. Importantly, sensory CIPN symptoms have previously been reported to begin first in the toes/feet and then move to the hands [57].

### Limitations 

This study provides new preliminary evidence of the impact of taxane chemotherapy on a variety of outcomes relating to neuropathy, HR-QOL, fatigue, pain, and physical functioning; however, there are several limitations. Firstly, the study included a small sample of participants, which means that caution is required when interpreting the results. Additionally, the study lacked ethnic and socioeconomic diversity as 100% of the participants were White British and were educated to at least lower general secondary education or vocational training. Different ethnic and socioeconomic groups may engage differently with this type of research and respond differently to cancer treatments. There is some evidence that African American patients have a markedly increased risk for both moderate and severe CIPN [89], and although this evidence was generated in the United States, it may indicate potential racial differences in the United Kingdom. Therefore, a lack of diversity in studies of neurotoxicity could mean that relevant risk factors, side effects and comorbidities are overlooked in sectors of the population, thereby increasing the health disparities gap.

Additionally, this study experienced some reduced data availability, particularly during and directly following chemotherapy. Missing data can reduce statistical power, cause bias in the estimation of parameters, and can reduce representativeness [90]. However, analysis via LMM was chosen as it can handle missing data under missing at random (MAR), without the need for imputations [51].

Participants received one of three regimens: (1) weekly paclitaxel alone, (2) anthracyclines followed by paclitaxel, or (3) anthracyclines followed by docetaxel. Therefore, participants in the study received different numbers of assessment points and a different number of chemotherapeutic agents. This is of even greater importance when considering the observed significant increases in patient-reported motor and autonomic symptoms following anthracyclines (post-anthracycline). This makes it challenging to fully understand the impact of the taxane chemotherapy alone, as any cumulative effects of the previous chemotherapy exposure must be considered.

Furthermore, almost all participants achieved the maximal score on the balance components of the physical performance battery at all time points, suggesting a possible ceiling effect. The SPPB, originally designed for older adults (65 and older) [91], may not have been sufficiently sensitive for this relatively younger cohort (mean age 50 ± 8 years), who demonstrated an adequate baseline level of physical function. As such, these findings may not be generalisable to older breast cancer populations, for whom the SPPB is more commonly validated.

Finally, for the peripheral nerve measures, it was judged as not feasible to mark recording and stimulating electrode placement. Although the differences in electrode placement between sessions would likely have been modest, an error this could have introduced should be considered when interpreting the results.

## Conclusion 

Self-reported and objective measures of CIPN, including assessments of postural sway and sensorimotor integration, were feasible to perform in this setting. Preliminary evidence indicated a concurrent increase in CIPN symptoms and postural sway, alongside reduced sensorimotor integration, in individuals receiving taxane-based chemotherapy. Following treatment, participants also reported increased fatigue and reduced HR-QOL. These findings provide a strong basis for future research with larger sample sizes and longer-term follow-up to better characterise CIPN trajectories. Measures of sensorimotor integration emerged as a particularly promising objective marker for CIPN.

## Supplementary Information

Supplementary Material 1.

## Data Availability

The datasets generated during and/or analysed during the current study are available from the corresponding author on reasonable request. The code used to analyse the data is available on Open Science Framework ( [https://osf.io/wfpv3] ).
